# Two Hop Adaptive Vector Based Quality Forwarding for Void Hole Avoidance in Underwater WSNs

**DOI:** 10.3390/s17081762

**Published:** 2017-08-01

**Authors:** Nadeem Javaid, Farwa Ahmed, Zahid Wadud, Nabil Alrajeh, Mohamad Souheil Alabed, Manzoor Ilahi

**Affiliations:** 1 COMSATS Institute of Information Technology, Islamabad 44000, Pakistan; farwaahmed17@gmail.com (F.A.); tamimy@comsats.edu.pk (M.I.); 2 University of Engineering & Technology, Peshawar 25000, Pakistan; zahidmufti@nwfpuet.edu.pk; 3 Capital University of Science and Technology, Islamabad 44000, Pakistan; 4 Biomedical Technology Department, College of Applied Medical Sciences, King Saud University, Riyadh 11633, Saudi Arabia; nabil@ksu.edu.sa (N.A.); salabed@ksu.edu.sa (M.S.A.)

**Keywords:** underwater wireless sensor networks, area towards destination, pipeline radius, virtual vector, potential neighbor number, vector-based forwarding, linear programming, composite priority function, qualified forwarding

## Abstract

Underwater wireless sensor networks (UWSNs) facilitate a wide range of aquatic applications in various domains. However, the harsh underwater environment poses challenges like low bandwidth, long propagation delay, high bit error rate, high deployment cost, irregular topological structure, etc. Node mobility and the uneven distribution of sensor nodes create void holes in UWSNs. Void hole creation has become a critical issue in UWSNs, as it severely affects the network performance. Avoiding void hole creation benefits better coverage over an area, less energy consumption in the network and high throughput. For this purpose, minimization of void hole probability particularly in local sparse regions is focused on in this paper. The two-hop adaptive hop by hop vector-based forwarding (2hop-AHH-VBF) protocol aims to avoid the void hole with the help of two-hop neighbor node information. The other protocol, quality forwarding adaptive hop by hop vector-based forwarding (QF-AHH-VBF), selects an optimal forwarder based on the composite priority function. QF-AHH-VBF improves network good-put because of optimal forwarder selection. QF-AHH-VBF aims to reduce void hole probability by optimally selecting next hop forwarders. To attain better network performance, mathematical problem formulation based on linear programming is performed. Simulation results show that by opting these mechanisms, significant reduction in end-to-end delay and better throughput are achieved in the network.

## 1. Introduction

In recent years, underwater wireless sensor networks (UWSNs) have gained much attention due to their growing application horizons like ocean exploration, disaster prevention, oil and gas extraction, technical surveillance for defense, etc. In underwater medium, acoustic waves are used as a means of communication instead of radio waves that absorb in water within the very short distance from the transmitter. Optical waves require alignment of the transmitter and receiver for link establishment. Therefore, acoustic communication is preferred for underwater wireless communication. However, the underwater acoustic communication faces unique challenges like limited available bandwidth, high end-to-end delay, severely impaired channel, etc. [1]. The network architecture of UWSNs comprises randomly-deployed sensor nodes equipped with acoustic links to communicate with each other and with the sink; whereas the sink is equipped with both acoustic and radio modems for the acoustic communication and the land communication, respectively. Sensor nodes report data to the sink, which are further offloaded to the monitoring center offshore.

The propagation speed of the acoustic signal is five-times less than the radio frequency, which results in higher end-to-end delays. Unstable network structure due to water currents in UWSNs is another challenge for traditional routing mechanisms to work in the underwater environment. Other factors like the low data rate are an outcome of limited available bandwidth of the acoustic channel. Similarly, severe channel impairments cause a high bit error rate. Both of these factors together affect the communication efficiency of UWSNs. The aforementioned reasons and constraints restrict the performance of conventional terrestrial schemes in the underwater environment. Moreover, the dynamic nature of UWSNs, where sensor nodes are mobile due to water currents may create void holes. Void hole creation may also occur due to energy fatigue of a sensor node. In these scenarios, if sensor nodes send data towards void regions, it may result in a packet drop that directly affects the packet delivery and energy consumption [2]. Therefore, throughput maximization has been extensively addressed in research because a high packet drop rate leads to high energy consumption. Hence, we need to apply throughput-focused routing techniques for energy conservation and void hole avoidance for reliable data delivery.

The proposed schemes, two hop adaptive hop by hop vector-based forwarding (2hop-AHH-VBF) and quality forwarding adaptive hop by hop vector-based forwarding (QF-AHH-VBF), aim to maximize network throughput and minimize delay. Firstly, the 2hop-AHH-VBF void hole avoidance scheme checks the void hole status of forwarding nodes before sending data packets. Secondly, the forwarding node selection is optimized to ensure reliable data transmission with minimized end-to-end delay particularly in local sparse regions. The other scheme, QF-AHH-VBF, formulates a composite priority function ($F_{p}$) after exchanging of the control packets in the data packet-forwarding stage. To carry out optimal forwarder selection at each hop throughout the routing path, every node calculates $F_{p}$. After relative comparison, the node with a high $F_{p}$ value is selected as the qualified forwarder. Afterwards, objective functions with linear constraints for either to maximize or minimize the performance parameters are defined under the linear programming-based problem formulation. As a final step, simulations are conducted to validate our schemes, which perform better than counter vector-based schemes in terms of successful transmissions and energy tax.

The remainder paper is structured as follows: state of the art work is categorized and tabulated in Section 2; Section 3 depicts a brief description of the network architecture following the subsections, problem definition and proposed schemes; in Section 4, the linear programming-based mathematical formulation is presented; simulation results in Section 5 are followed by Section 6 and Section 7, concluding the paper and discussing trade-off and conclusion, respectively.

## 2. Related Works

From the existing works, routing protocols and schemes in wireless sensor networks (WSNs) are referred with brief descriptions of their features, advantages and limitations in the current section. Besides, the routing protocols related to the proposed schemes are also reviewed at the end of this section. For the sake of simplicity, related works are summarized in Table 1.

Routing strategies implemented in terrestrial WSNs (TWSNs) are unable to perform effectively in the detrimental acoustic environment even if the metrics are well configured [3]. The major reason behind the lack of performance of the terrestrial-based routing mechanism is the unique nature of the acoustic channel, like the limited bandwidth, high delays, the mobility of sensing devices due to water currents, etc. [4]. For the sake of simplicity and better understanding, some of the TWSN routing protocols are discussed as two categories: proactive routing and reactive routing. Unfortunately, neither of the categories is suitable for the dynamic underwater environment due to the large overhead of control messages exchanged at the setup of the network and whenever the topology changes due to water currents. Due to the high exchange of control packets in proactive routing, nodes’ energy dissipates quickly, resulting in void holes. Optimized link state routing protocol OSLR [5] and destination sequenced distance vector protocol DSDV [6] are proactive routing protocols having a high energy consumption due to large overhead, which ultimately degrades the network performance. Similarly, the use of reactive routing protocols is relatively better than proactive strategy, but the delay is very high as the acoustic channel already suffers from high delay. That is accepted for a dynamic situation when the established links between the source and destination are symmetric. Due to the high dynamic nature of the acoustic channel, asymmetric links are setup. Therefore, reactive routing protocols (Ad hoc on-demand distance vector AODV [7], Dynamic source routing DSR [8], etc.) yield high latency and are not well suited for the acoustic medium. The difference between the UWSN and TWSN protocols is given in Table 2.

Geographical routing techniques have the ability to minimize the end-to-end latency and overhead, where information is not flooded between the source and destination. However, information regarding the location of the neighbor and destination is utilized for transmission. The limitation for the adaptation of this routing mechanism is that the global positioning system (GPS) does not work well due to high absorption and quick attenuation of the signal in the acoustic medium. While keeping in mind the aforementioned constraints, the research community has been devoted to bringing continuous improvement of underwater protocols. Keeping different considerations into account, routing protocols for UWSNs are classified into beacon-based routing, clustering routing, depth-based routing and location-based routing protocols. The hop by hop dynamic addressing-based (H2-DAB) protocol does not keep location information of the nodes. Unique ID assignment to every node is performed based on hop counts from the sink. Reliable data delivery is achieved, compromising the network overhead [9]. In cluster-based routing [10,11], cluster heads are selected based on residual energy or location. Features like efficient network management and communication overhead minimization are achieved. Due to longer end-to-end delays, it gives poor performance in time-critical applications. In the depth-based routing (DBR) scheme, nodes greedily forward data packets to the upper nodes. If it does not find the next hop forwarder node due to a coverage hole or energy hole, packet drop occurs, which affects packet delivery in DBR [12]. In the energy-efficient depth-based routing protocol (EE-DBR), energy conservation is obtained based on depth information. It incurs low end-to-end delay and less energy consumption compromising the network overhead [13]. The weighting depth and forwarding area division depth-based routing (WDFAD-DBR) protocol addresses the problem of void holes in UWSNs. In WDFAD-DBR, the extra energy expenditure and average end-to-end delay are reduced by suppressing duplicate packets in dense regions. Thus, enhanced reliability and reduced energy expenditure are achieved. In WDFAD-DBR, routing decisions are based on two metrics: firstly, the depth of both current nodes and the expected next hop forwarder are considered. Secondly, WDFAD-DBR adjusts the forwarding area according to the channel condition and node distribution density. WDFAD-DBR has achieved a lessened probability of void holes; however, considering the depth difference between two hops to avoid void holes does not eliminate them effectively, and eventually, packet drops affect the network lifetime [14]. Similarly, in [15], interference-aware routing protocols (Intar: interference-aware routing; Re-Intar: reliable and interference-aware routing) are presented. These protocols have adopted the sender-based approach to avoid void holes in UWSNs. The proposed protocols improve void hole avoidance along with collision probability at the receiver side; thus, the packet delivery ratio is improved. The Intar scheme computes the cost function (CF) based on multiple network parameters in the forwarding phase. The sender node selects the next forwarder on the basis of CF. The potential forwarding node with the least number of neighbors, the minimum number of hops from the sink and the maximum distance from the sender node computes the maximum value of the CF function. This approach reduces collision probability and avoids void holes by choosing the potential forwarding node with the maximum CF value. In Re-Intar, CF is computed using the depth difference between the source node and the potential forwarding node along with other parameters taken from the Intar protocol. For successful transmission, the source node transmits one hop backward in the case of the void region at the cost of increased accumulative propagation distance.

In location-aware vector-based forwarding (VBF), the forwarding area for transmission is confined within a virtual pipeline that is directional towards the destination. The virtual pipeline direction and radius remain constant from a source to a destination. In sparse networks, the unavailability of forwarder nodes due to the constant virtual pipeline radius causes retransmissions. Therefore, the VBF scheme does not perform well in sparse networks [16]. The performance of the VBF scheme is improved in the hop by hop VBF (HH-VBF) protocol by changing the direction of the virtual pipeline hop by hop. However, the unchanged pipeline radius restricts the network performance in the sparse node deployment because the probability of the void hole is not eliminated effectively. In the case of a small pipeline radius, the current forwarding node does not find potential forwarders in its effective transmission range. In other case, when the pipeline radius is large, packet duplication occurs due to the large number of forwarders in the pipeline [17]. This problem is further optimized in adaptive hop by hop vector-based forwarding (AHH-VBF). In [18], the pipeline radius (PR) and transmission radius (TR) are adaptively changed hop by hop taking the neighbor node distribution into account. Moreover, the adaptive transmission radius is intended to avoid unwanted energy consumption. It is noteworthy that we have taken the motivation from the AHH-VBF scheme. In this paper, 2hop-AHH-VBF and QF-AHH-VBF adopt the adaptive PR and TR features of AHH-VBF; however, the proposed schemes are different in the following aspects.

1. There is an inefficient void hole avoidance mechanism opted for in AHH-VBF that is further modified in the proposed scheme.
2. Forwarder selection in 2hop-AHH-VBF is based on the potential neighbor number (PNN) of the forwarder node in the area towards the destination (ATD). Hence, it aims to optimize the forwarder selection criterion different from the AHH-VBF adopted.
3. In QF-AHH-VBF, every node computes the composite priority function ($F_{p}$) to elect itself as a qualified node. The node that secures high $F_{p}$ among the PNN of a sender node is assigned the lowest holding time. Eventually, network throughput and end-to-end delay are achieved opting for this mechanism.

## 3. Proposed Schemes

This section describes the proposed schemes (2hop-AHH-VBF and QF-AHH-VBF) following the network model and problem description.

### 3.1. Network Architecture

**Figure 1:**
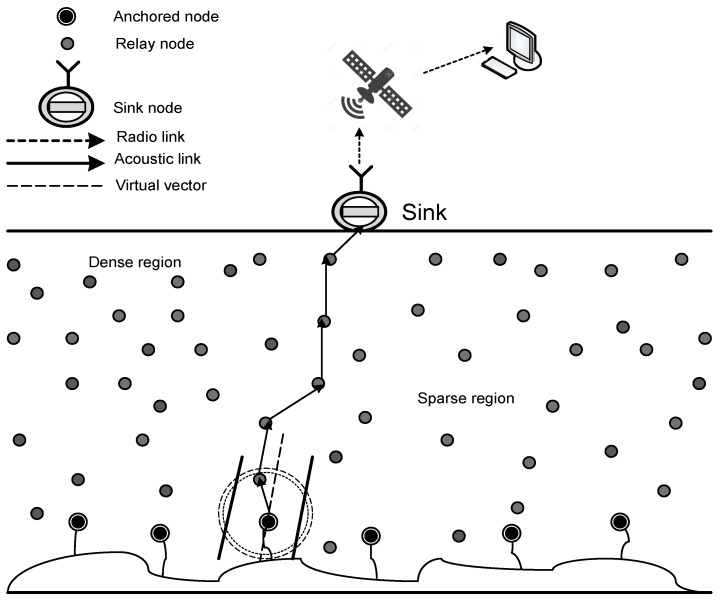
Network architecture.

In the network architecture, $N=n_{1},n_{2},n_{3},...,n_{n}$ sensor nodes are deployed randomly in a three-dimensional area. Sensor nodes could be of different types in terms of size, battery, processing capabilities, etc. [19,20]. This network consists of homogeneous sensor nodes and a sink deployed at the ocean surface as shown in Figure 1. All of these sensor nodes are equipped with sensing devices and an acoustic modem for communication purposes. Sensor nodes are deployed at different depths, and data are relayed towards the destination node using forwarding mechanisms. Data are reported to the sink, which is equipped with an acoustic modem and a radio modem. Acoustic links enable it to connect with sensor nodes, and radio links are used for the land communication. Received data at sink node are further transmitted towards satellites using radio links. At the end of this forwarding process, data reach the data center onshore.

### 3.2. Problem Definition

**Figure 2:**
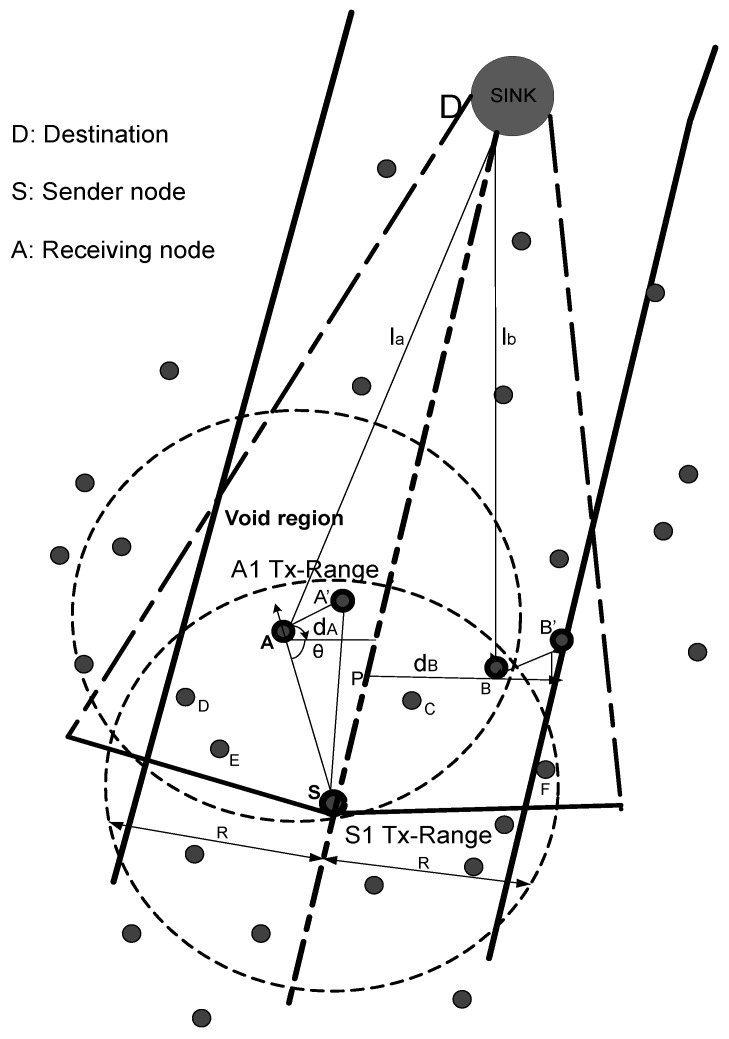
Problem identified regarding forwarder selection.

In UWSNs, the frequent mobility of sensor nodes results in the non-uniform distribution of nodes. There are some local regions with high node density, and others are low node density regions, as shown in Figure 1. Low node density regions become sparse due to high node mobility and the void regions created. That is caused due to two possibilities: either the unavailability of sensor nodes in a region or due to the energy hole that creates the coverage hole. This problem leads to high energy consumption due to packet drops. In AHH-VBF, the forwarder node selection on the basis of the relative position to the virtual vector (desirableness factor) and the maximum distance from the source node does not avoid void holes effectively. For example, as in Figure 2, Node S is the current forwarding node, and a circle named S1 Tx-range is the transmission range of Node S. Two parallel lines shaped as are virtual pipeline restrict transmissions within this area. Nodes A, B, C, D, E and F are above Node S in its upper hemisphere of transmission range. Some nodes are suppressed neighbors below Node S. When Node S sends a packet, Nodes A, B, C, D, E and F along with suppressed nodes receive the packet. Suppressed nodes discard the packet directly, whereas Node F also discards the packet because it is outside of the pipeline. In AHH-VBF, Node S selects Node A on the basis of the relative position of Node A with respect to the virtual vector, and its distance is the maximum from Node S; whereas Nodes B, C, D and E are distant from the sink as compared to Node A. Therefore, these nodes are not qualified for forwarding node selection. However, the selection criteria for forwarding the node within the one-hop neighbor range is not efficient enough to cater to void hole problem. As illustrated in Figure 2, the void region is created due to the unavailability of nodes above Node A in the A1 Tx-range. If Node A continues to forward the packet in the void region, the packet drop occurs. Hence, the probability of unsuccessful transmissions in this scenario shows that forwarding node selection needs to be optimized to improve packet delivery.

### 3.3. Two-Hop Adaptive Routing Scheme

Energy is one of the most scarce resources in sensor-based networks. There are various reasons behind the inefficient depletion of nodes’ energy, which degrade the network performance. One of the major concerns is the void hole. The occurrence of the void hole consumes unnecessary energy and stops the flow of data between the source and destination due to the breakage of routes and the unavailability of forwarder node(s). Instead of recovering from the void hole, we have proposed a mechanism through which the creation of the hole is avoided prior. For catering to the void hole problem, four parameters are taken into consideration. These parameters are: the residual energy of the forwarder node, the maximal distance between the sender and receiver within the communication range, a threshold value for the number of nodes to be in the pipeline for minimizing retransmissions and the distance of the nominated forwarder node from the virtual vector. The reason behind the computation of the aforementioned parameters is to ensure that the forwarder node is not selected repeatedly, because it will dissipate the node energy quickly and the void hole will be created. Therefore, with effective forwarder node selection, an efficient battery dissipation is ensured. Energy computation at each hop is performed to accommodate any of the changes in the function for suitable node selection as forwarder for optimal results in terms of network performance.

**Figure 3:**
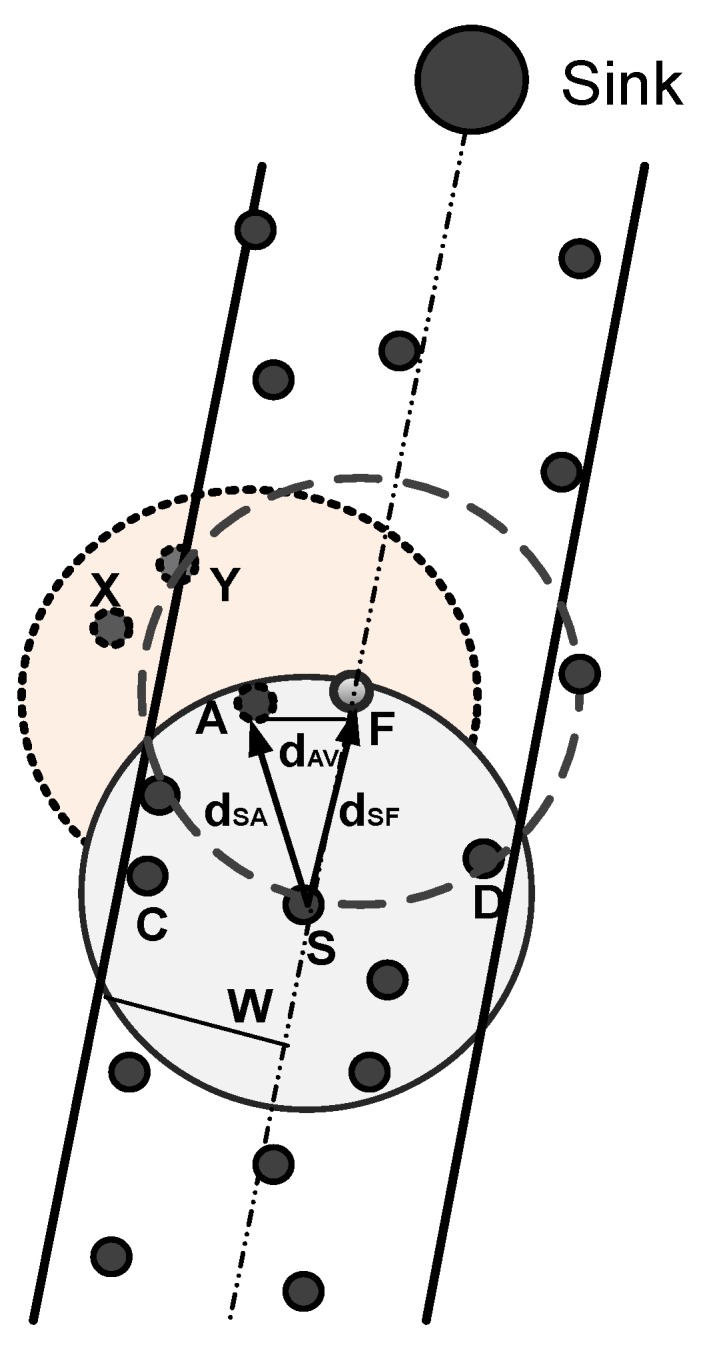
Illustration for holding time.

The transmission within the communication range means the signal to noise ratio is good; therefore, we have preferred a next neighboring node at optimal distance from the destination of sender node because the signal is broadcast within the transmission radius, which means the energy will be dissipated, so the node at the optimal distance is the priority. Similarly, the forwarder node having one neighbor is avoided due to node mobility. If more than one node is in the transmission range, the number of re-transmissions is reduced, and data packets reach the destination with low latency. Furthermore, the distance from the virtual vector accounts for if the data are forwarded before they reach the destination, and the neighbor node should be in the communication by keeping in view the mobility of the node due to water currents. All metrics are obtained at each up to two hops before nominating a forwarder node in order to minimize the void hole occurrence probability. The probability that the void hole is successfully detected and avoided can be computed as $\left(1-{\left(1-p\right)}^{2}\right)$, where *p* is computed by $p=\frac{\pi r^{2}}{R}$. Here, *r* denotes the sensing range of a sender node, while *R* represents the radius of the network. Due to the reception of duplicate data packets, much energy is wasted. Therefore, the holding time is computed based on the number of eligible forwarder nodes in the sensing range (Figure 3). The following equations are formulated to show that redundant transmissions are avoided for network lifetime prolongation.

(1) $$Thold_{2hop}=\frac{\alpha \times T_{pre}}{2\times f_{Nn}}+\frac{R_{t}-\left|SF\right|}{V_{sound}}$$

where $f_{Nn}$ is computed as given in Equation (2).

(2) $$f_{Nn}=\frac{1+sign(Nn-2)}{2},$$

sign(Nn−2) is calculated according to the following equation.

(3) $$sign\left(Nn-2\right)=\left\{\begin{array}{cc} -1, & Nn<2\\ 0, & Nn=0\\ 1, & Nn>2 \end{array}\right.$$

To ensure that each parameter has an equal contribution to selecting the potential forwarder node, a mathematical formulation is carried out in the following section.

### 3.4. Quality Forwarding for Forwarder Node Selection

Forwarding is performed for routing data packets towards the destination. There are different kinds of forwarding, such as selective diversity forwarding (SDF), next hop forwarding, host-specific forwarding, etc. [4]. Our forwarding is similar to SDF; however, we compute multiple parameters for selecting the next forwarder node. To illustrate the changes of parameters for forwarder node selection in the defined function, a mathematical formulation is carried out via taking into account: the residual energy of the forwarder node, the number of potential neighbor nodes, the distance between the sender and forwarder node and the distance of the forwarder node from the virtual vector for handling the mobility factor due to water currents. The composite function is defined in Equation (4).

(4) $$f_{5}=f_{1}\times f_{2}\times f_{3}\times f_{4}$$

where, $f_{1},f_{2},f_{3},\mathrm{and}\;f_{4}$ are computed as follows:

(5) $$f_{1}=E_{r},\;E_{r}\in \left[E_{tx}\;E_{o}\right]$$

Fitness function $f_{1}$ shows that it is directly proportional to the residual energy of a node. The initial energy provided to every node is 100 J; therefore, the residual energy of a node is always equal to or less than $E_{o}$.

(6) $$f_{2}=1+sign\left(Nn-2\right),\;N_{n}<=4$$

$f_{2}$ is based on the threshold value to ensure that a forwarder should have potential forwarders equal to or greater than the threshold value.

(7) $$f_{3}=D_{ij},\;D_{ij}\in \left[0\;D_{max}\right]$$

$f_{3}$ depicts that the distance between the sender and receiver node is directly proportional to the energy consumption. This function provides the distance between the sender node and the forwarder, for which the larger the better. If the distance between the sender node and the forwarder is large, the packet will traverse few hops during the transmission. The objective is to minimize the number of hops for qualified forwarder selection for minimizing the energy consumption.

(8) $$f_{4}=1/\left(1+d\right),\;d\in \left[0\;R_{t}/2\right]$$

$f_{4}$ denotes the distance of the forwarder node from the virtual vector. The smaller the distance, the higher will be the chances of void occurrence. The computation of this parameter is specifically to ensure that the node must be within the sensing range if data are transmitted to it.

By means of normalization, we have computed the value for the proposed composite function within interval [0, 1]. This is intended to avoid undue favor for any of the aforementioned parameters, while computing $F_{p}$ for a qualified forwarder node.

(9) $$F_{p}=\frac{\left(1+Sign\left(x-2\right)\right)\times D_{ij}\times E_{r}}{2\times \left(1+d\right)\times D_{max}\times E_{o}}$$

Once the composite function is calculated, now potential forwarder nodes are nominated. In order to find out the optimal holding time for each qualified node $F_{p}$, the following equation is used.

(10) $$Thold_{QF}=T_{pre}\times F_{p}+\frac{R_{t}-\left|SF\right|}{V_{sound}}$$

### 3.5. Packet Types

Before proceeding to the packet types used during the network operation, some of the definitions need to be described for avoiding ambiguities.

Area towards the destination (ATD): This is an area where the neighbors of the sender node are closer to the destination.

Potential neighbor number (PNN) is defined as nodes deployed in ATD are nearer to the destination than the sender node. In order to initiate the network process, a few control messages need to be exchanged among the network nodes to allow the communication between the network nodes. After the deployment of the node, for establishing routes between and the destination, neighbor query (NQ) and neighbor Ack (NA) packets are exchanged. NQ is broadcast by the sender node to find out PNN in ATD. The format of NQ is a tuple (type ID, sender ID, SX, SY, SZ, PNQ where type ID is the type of packet and its value is “00”, sender ID denotes the address of the node sending NQ and SX, SY, SZ are the *x*-coordinate, *y*-coordinate and *z*-coordinate of the sender node, respectively. To reduce the overhead of the control message, Boolean values are used to represent the types of packets. NA packets are sent back from neighbor nodes to the sender node after receiving NQ. The NA format has three fields (type ID, sender ID, X, Y, Z, $PN_{n}$), where type ID is “01” that shows NA, sender ID is the address of the node sending NA and X, Y, Z are the *x*-coordinate, *y*-coordinate and *z*-coordinate of the sender node, respectively. The data packet header format consists of (type ID, sender ID, destination ID, SX, SY, SZ, FX, FY, FZ, PID, PR, TR where PID is packet ID, PR, TR is pipeline radius and transmission radius, respectively.

**Figure 4:**
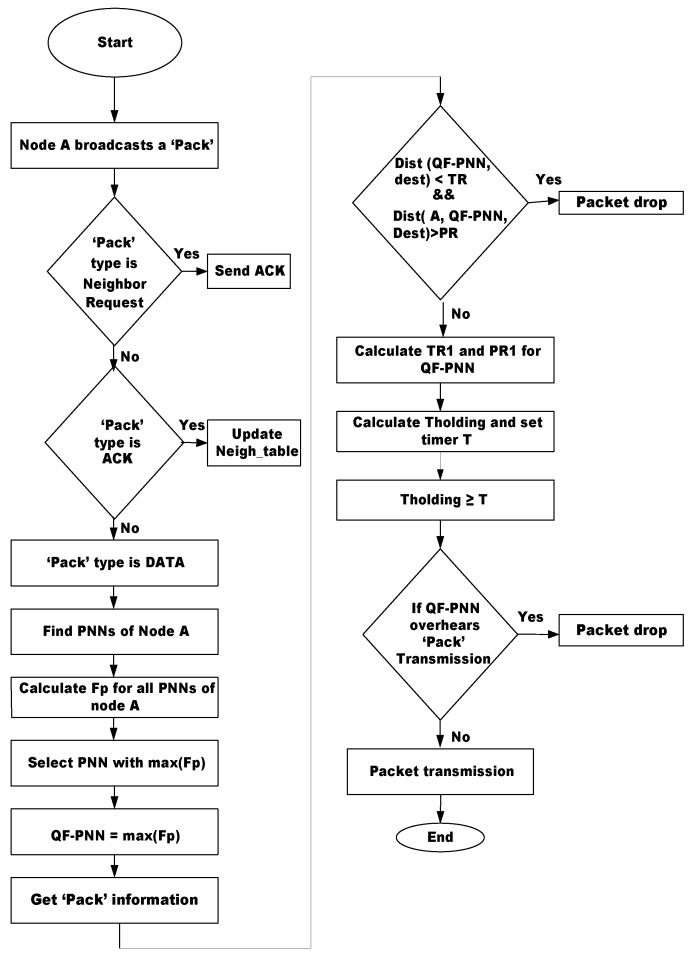
Algorithm for QF-AHH-VBF.

These fields have the following meanings: sender ID is the ID of the broadcasting node; destination ID shows the address of the destination node; SX, SY, SZ and FX, FY, FZ are the *x*-coordinate, *y*-coordinate and *z*-coordinate of the sender node and the forwarding node, respectively. The PSN field is for packet sequence number, and PR and TR show the pipeline radius and transmission radius, respectively. The message exchange procedure repeats when the void hole occurs; else, the same routes are used. The unnecessary use of the control message is avoided, which ensures that the energy dissipated is minimal, resulting in efficient network performance. The changes in the function are handled through the composite function defined in Section 3.4. The algorithm of proposed scheme is presented in Figure 4.

## 4. Linear Programming-Based Mathematical Formulation

Linear programming is an extensively-used mathematical approach to attain an optimal outcome. Initially, an objective function either tends to maximize or minimize and is formulated with linear constraints. We have used the simplex method of linear programming to find out feasible regions for performance parameters. In this section, it is discussed how linear programming-based problem formulation helps with the maximization or minimization of performance parameters in order to improve the network performance.

### 4.1. Energy Tax Minimization Using Linear Programming

High energy consumption drastically affects network performance due to the confined power resource in UWSNs. Many routing protocols have addressed this problem. For the sake of energy tax minimization, an objective function with multiple linear constraints is formulated. In QF-AHH-VBF, the energy tax of sensor nodes mainly depends on packet transmission and packet reception between a source node and the sink. Thus, keeping this in mind, we have formulated an objective function for energy tax minimization in Equation (11).

(11) $$Min\sum_{i=1}^{N}E_{Tax}\left(i\right)\;\;\forall \;i\;\in \;N$$

where $E_{Tax}$ is energy tax calculated for all nodes.

(12) $$E_{consumed}\left(ij\right)=\sum_{i=1}^{N}\left(E_{TX}\times D_{\left(ij\right)}+E_{RX}\times N_{n}\right)\;\;\forall \;i,j\;\in N$$

where Nn≥0 and Dij≥0. In Equation (12), $E_{consumed}$ mainly depends on the transmission energy of the sender node $E_{TX}$ consumed over the distance $\left(D_{\left(ij\right)}\right)$ between the sender node and the receiver node. The receiving energy $\left(E_{RX}\right)$ is consumed due to number of neighbor nodes $\left(N_{n}\right)$ of the sender node receiving the packet.

(13) $$E_{TX}^{max}=P_{TX}\times \left(HS+PL\right)/DR$$

(14) $$E_{RX}^{max}=P_{RX}\times \left(HS+PL\right)/DR,$$

Equations (13) and (14) give the maximum values of $E_{TX}$ and $E_{RX}$. These values depend on the transmission power required for the data packet size (HS + PL) as per the data rate (DR).

(15) $$E_{total}=E_{initial}\times N$$

$E_{total}$ is the total energy provided to all of the nodes in the network as initial energy $\left(E_{initial}\right)$ in Equation (15). Energy tax is basically the amount of energy consumed in all of the simulation rounds, which is stated in Equation (16) as:

(16) $$E_{Tax}=\sum_{r=1}^{r_{max}}\left(E_{consumed}\left(r\right)\right)$$

Subject to:

All of the linear constraints of the objective function are given in Equations (17)–(20).

(17) $$E_{\left(TX,RX\right)}\leq {E_{i}}^{initial}\;\;\forall \;i\;\in \;N$$

$E_{\left(TX,RX\right)}$ is the energy required for the transmission and reception collectively, which should be less than the initial energy provided to a node. Meanwhile, Equation (18) states that $E_{\left(TX,RX\right)}$ should be greater than or equal to the residual energy of a node.

(18) $$E_{\left(TX,RX\right)}\geq {E_{i}}^{r}\;\;\forall \;i\;\in \;N$$

In Equation (19), the distance between a sender node and a receiving node should always be less than or equal to the transmission range of the sender node $R_{TX}^{max}$. Similarly, this distance must not be equal to zero, which is shown as $R_{TX}^{min}$ in Equation (20),

(19) $$D_{i}^{j}\leq R_{TX}^{max}\;\;\forall \;i,j\;\in \;N$$

(20) $$D_{i}^{j}\geq R_{TX}^{min}\;\;\forall \;i,j\;\in \;N.$$

(21) $$E_{TX}^{min}=E_{TX}/L$$

(22) $$E_{RX}^{min}=E_{RX}/L$$

Graphical analysis: Assume a scenario in which the transmission range is 2000 m, which is divided into *L* levels, i.e., *L* = 1, 2, 3, 4 and 5. Dividing the transmission range into levels is intended to observe the energy consumption according to these levels as written in Equations (25) and (26), where HS + PL = 888 bits, DR = 16 kbps, N = 500, $P_{TX}$ = 50 W and $P_{RX}$ = 0.158 W, respectively. From these parameters, $E_{TX}$ is 4.995 J calculated from Equation (25) at *L* = 1 and 0.999 J from Equation (25) at *L* = 5. By Equation (26), $E_{RX}$ is 0.555 J calculated at *L* = 1 and 0.111 J calculated at *L* = 5, respectively.

(23) $$1\leq E_{TX}+E_{RX}\leq 5.550$$

(24) $$0.999\leq E_{TX}\leq 4.995$$

(25) $$0.111\leq E_{RX}\leq 0.555$$

**Figure 5:**
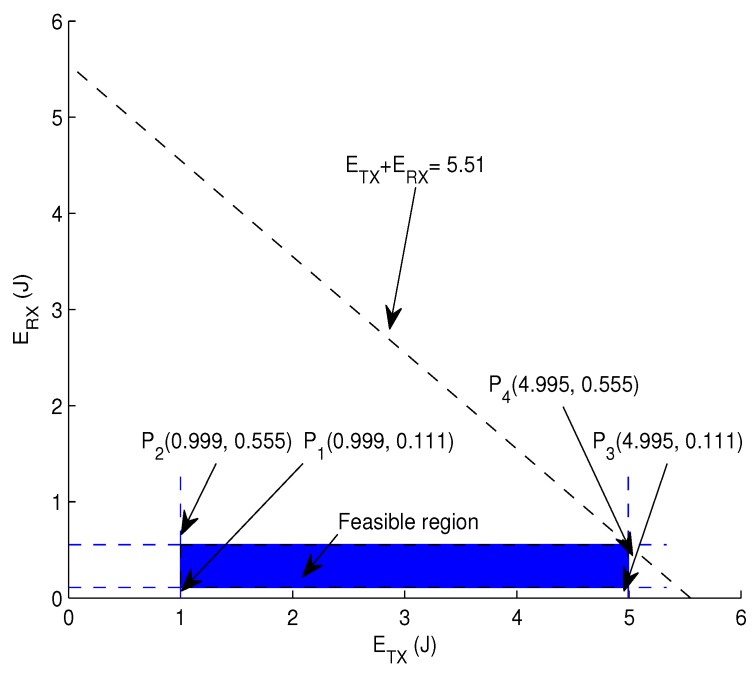
Feasible region for energy tax minimization.

Corresponding to the bounds mentioned above, Figure 5 depicts the intersection region in which all feasible solutions lie. This bounded region is named the feasible region. Any point laying within the bounded region yields a valid solution.

As the next step, we test each vertex depicted in Figure 5 as:

at $P_{1}:0.999+0.111=1.110$ J
at $P_{2}:0.999+0.555=1.554$ J
at $P_{3}:4.995+0.111=5.120$ J
at $P_{4}:4.995+0.555=5.550$ J.

Hence, this method provides a valid solution satisfying these bounds. Therefore, the values of transmission and reception energy within the feasible region tend to minimize the energy consumption for the optimal solution.

### 4.2. End-To-End Delay Minimization Using Linear Programming

Graphical analysis: Assume a scenario in which if the sender node is in the transmission range of the sink, it transmits directly; whereas, if the sink is a few hops away from the sender node, the data packet is sent over multiple hops. Consider the one-hop transmission delay as the minimum and delay incurred by multiple hops the maximum delay due to multiple hops. In the direct transmission scenario, we divide $D_{SD}$ into *L* levels, i.e., *L* = 1, 2, 3, 4 and 5.

$5.83\leq D_{DT}+D_{MHT}\leq 11.83$
$1.888\leq D_{DT}\leq 3.951$
$2.949\leq D_{MHT}\leq 7.888$

**Figure 6:**
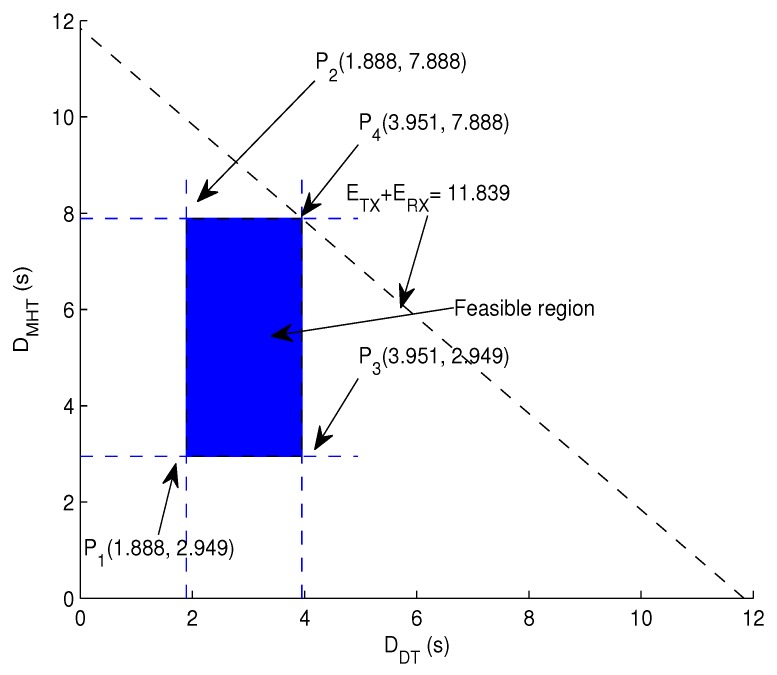
Feasible region for end-to-end delay minimization.

Each vertex of the region shown in Figure 6 is as:

at P1:1.888+2.949=4.873s
at P2:1.888+7.888=9.776s
at P3:3.951+2.949=6.9s
at P4:3.951+7.888=11.839s

(26) $$Min\sum_{i=1}^{N}D_{tot}\left(i\right);\;\;\forall \;i\;\in \;N$$

(27) $$D=D_{TX}+D_{Prop}+T_{hold}$$

(28) $$D=D_{SD}/V_{sound}+\left(HS+PL\right)/DR+T_{hold}$$

(29) $$D_{tot}=D_{DT}+D_{MHT}$$

(30) $$D_{DT-min}=D_{DT}/L;$$

(31) $$D_{MHT-min}=H_{n-min}\times D;$$

(32) $$D_{MHT-max}=H_{n-max}\times D;$$

where $T_{hold}$ is taken from Equation (10).

at C1: $0<D_{SD}\leq R_{TX}$
at C2: $H_{n-min}\leq H_{n}\leq H_{n-max}$
at C3: $T_{hold-min}\leq T_{hold}\leq T_{hold-max}$

### 4.3. Throughput Maximization Using Linear Programming

**Figure 7:**
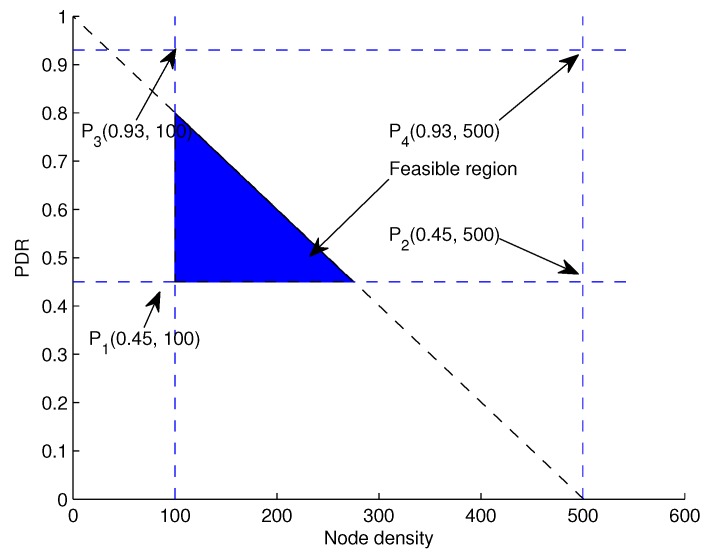
Feasible region for throughput maximization.

The objective function is formulated to maximize the network throughput considering the minimization of energy consumption. In our proposed schemes (2hop-AHH-VBF and QF-AHH-VBF), data packets are relayed using multi-hoping mechanism. The network throughput is the total number of data packets received successfully at the sink. Considering link quality for successful transmission, δ is defined as the threshold value for the neighbor number of a sender node. The link quality check is to ensure successful packet delivery. Moreover, the energy required for transmitting a packet should be less than the residual energy of a node participating in the forwarding process as stated in C1. The distance between a pair of sender and receiver nodes must be greater than $D_{ij}^{min}$ and less than $D_{ij}^{max}$ as in C4. All of these linear constraints are taken into consideration while formulating an objective function represented in Equation (33),

(33) $$Min\sum_{i=1}^{N}T_{p}\left(i\right);\;\;\forall \;i\;\in \;N$$

(34) $$Min\sum_{r=1}^{r_{max}}T_{p}\times P;\;\;\forall \;i\;\in \;N,$$

such that:

C1:$E_{TX,RX}\leq E_{r}$
C2:$P_{link}\geq \delta$
C3:$E_{TX,RX}\geq E_{th}$,

where $E_{th}$ is the residual energy required for transmission and reception:

C4:$0<D_{ij}\leq D_{ij}^{max}$. Feasible solution lies in the shaded region as can be seen in Figure 7.

## 5. Performance Evaluation

This section contains the simulation analysis of the proposed schemes followed by the simulation setup and results. One hundred to 500 sensor nodes are deployed in a three-dimensional area of 10 km × 10 km × 10 km, and the sink is fixed on the surface. The maximum power for transmission and reception is 90 dB re μ Pa and 10 dB re μ Pa, respectively. The maximum transmission range is selected as 2000 m, because as in [9], an increase in transmission range does not affect network performance. To stop node movement outside the region, the random walk 2D mobility model is used. Each sensor moves at a speed of 1–3 m/s. Vertical movement is considered negligible, whereas horizontal movement in the X-Y plane is commonly considered in the underwater environment. The header size and data size of the data packet are 39 bytes and 72 bytes, respectively. Neighbor query and neighbor acknowledgment packets have 66 bits and 114 bits, respectively. In each round during the simulations, the simulation results were run over 50 times, and each time, the network topology was random. For simplicity, simulation parameters are tabulated in Table 3.

Due to the detrimental characteristics of the aquatic environment including, but not only, ones having a limited bandwidth, high propagation and high attenuation, the MAC protocol is not very effective for identifying and avoiding collisions in the network [21]. In order to mitigate the aforementioned constraints, pure ALOHA is one of the widely-accepted protocols due to its insensitivity for long propagation delay [22]. Therefore, we have adopted pure ALOHA for our simulation results.

### 5.1. Performance Metrics

Performance metrics used in this paper are: energy tax, packet delivery ratio (PDR), end-to-end delay and accumulated propagation distance (APD).

Energy tax: average energy consumption per node while a packet successfully reaches the destination. Energy consumption includes energy consumed in transmission and reception of a packet and exchanging control packets. The mathematical notation for this metric can be written as:

(35) $$EnergyTax=\frac{E_{t}}{NodeNum\times packets}.$$

where $E_{t}$ total energy consumption of the network is divided by the number of nodes deployed and the number of packets successfully reached at the sink. Energy tax is measured in joules.

PDR: ratio of packets successfully received at the sink to packets sent by the sender node.

End-to-end delay: average time taken in transmitting packets from the sender node to successfully being received by the sink. It includes propagation delay, transmission and reception delay, holding time and calculation time taken in a successful transmission to the sink. End-to-end delay is measured in seconds.

APD: the average propagation distance traveled at every hop traversed for all of the packets successfully reaching the sink. This is measured in km.

### 5.2. Discussion

A comparative simulation analysis of the proposed schemes with respect to the existing scheme AHH-VBF is discussed in this section. The discussion is based on the comparison of the aforementioned performance metrics.

### 5.3. Packet Delivery Ratio

**Figure 8:**
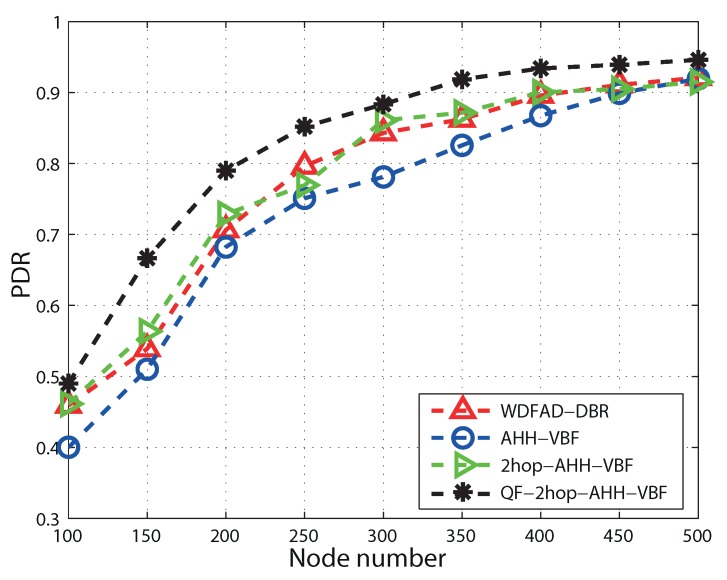
Packet delivery ratio comparison.

It can be seen in Figure 8 that as the node number increases, PDR begins to increase in all of the schemes. This is because the PNN per node increases as the node number increases. Hence, the void hole probability reduces due to the availability of qualified nodes in the ATD. PDR increases till a certain threshold; after that, collision at the receiver results in reduction of PDR. Below 300 nodes, 2hop-AHH-VBF and QF-2hop-AHH-VBF perform better than AHH-VBF due to the optimal forwarder selection in the ATD. This selection ensures successful packet transmission even in sparse regions in the network. As the node number increases beyond 300 nodes, the difference of PDR of all of the schemes reduces due to the fact that the increase in the node number itself reduces the void hole probability. However, QF-2hop-AHH-VBF outperforms AHH-VBF because of an optimal forwarder selection criterion in terms of node position and residual energy. Hence, QF-2hop-AHH-VBF performs better in sparse and dense environments. In dense network regions, duplication of packets is handled with the constrained pipeline radius as set by the AHH-VBF scheme. QF-2hop-AHH-VBF performs 5.6% better than AHH-VBF, while 2hop-AHHH-VBF and WDFAD-DBR perform slightly better than AHH-VBF in sparse regions. In dense regions, duplication of packets is avoided by opting for the mechanism to restrict the forwarding area in all four schemes. Hence, the performance pattern of all of the schemes is similar for a high number of nodes.

### 5.4. Energy Tax

**Figure 9:**
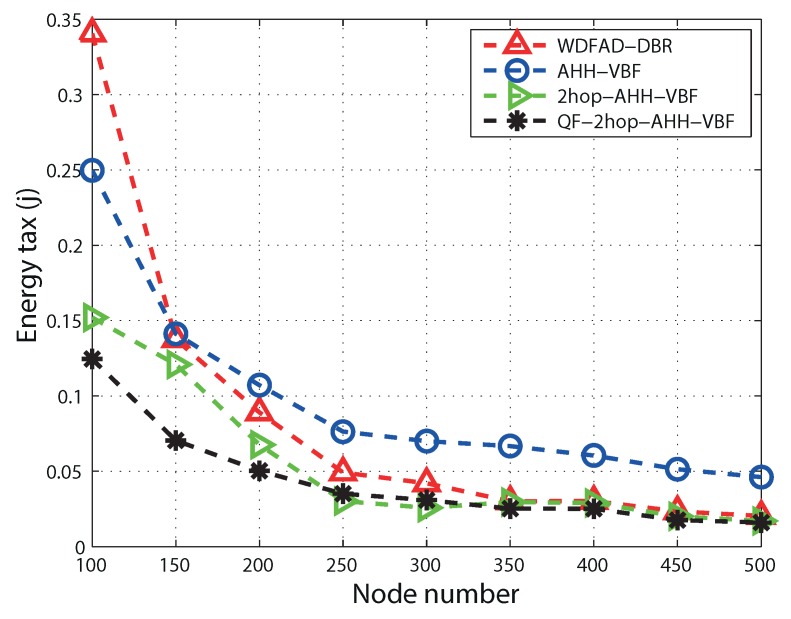
Energy tax comparison.

Energy tax tends to decrease with the increase in node number in all four schemes. This is due to the fact that the increase in node density increases the number of successful transmissions. In low node density regions, packet drop due to the unavailability of forwarding nodes results in more energy consumption. As in Figure 9, the AHH-VBF scheme takes more energy, while the node number is less. In the AHH-VBF scheme, information of the next hop forwarding node is not considered while transmitting the packet. Therefore, it leads to packet transmission towards the void region, and unnecessary energy tax is consumed due to transmission failure. It can be seen that the 2hop-AHH-VBF scheme consumes less energy than the AHH-VBF scheme because the unsuccessful transmissions are avoided here. While node number increases beyond 300, the difference of energy consumption reduces. QF-2hop-AHH-VBF performs better in terms of PDR; hence, it takes more energy consumption due to successful transmissions towards the destination. Moreover, the selection of the quality forwarder in QF-2hop-AHH-VBF is based on the two-hop information and the residual energy of forwarding nodes. Optimized selection of the qualified node for the forwarding process minimizes network overhead due to control packets. Hence, initially, the energy consumption of the QF-AHH-VBF scheme is high because of high PDR when compared with other routing schemes. Gradually, the energy tax difference is observed as the node number increases beyond 400. Similarly, QF-AHH-VBF outperforms WDFAD-DBR because the constrained pipeline radius avoids packet duplication, and qualified node selection based on the composite function provides the optimal path towards ATD. WDFAD-DBR decides the path on the basis of the weighting depth difference, which does not completely avoid packet duplication. Thus, WDFAD-DBR has unnecessary energy consumption.

### 5.5. End-To-End Delay

**Figure 10:**
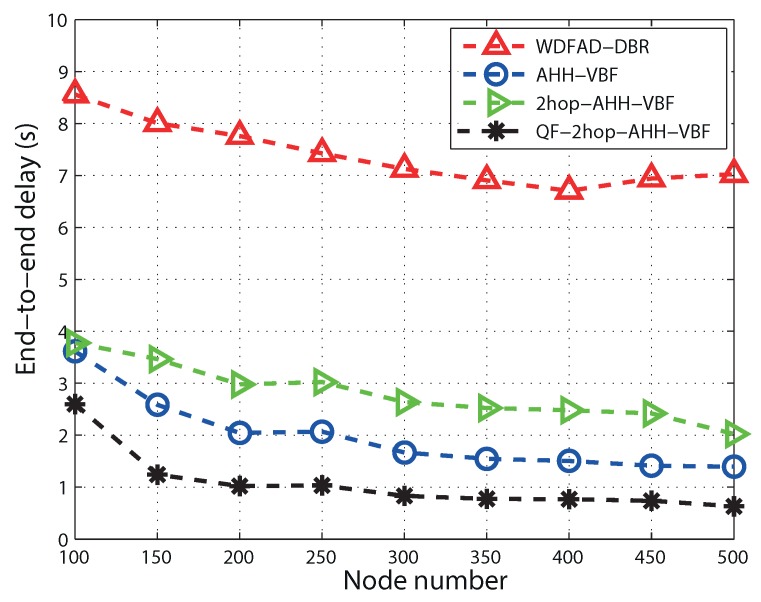
End-to-end delay comparison.

**Figure 11:**
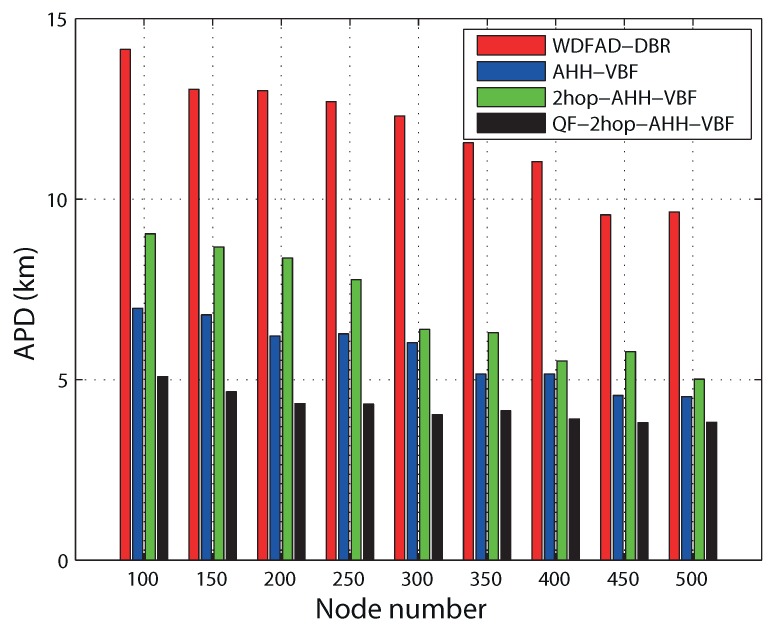
Accumulative propagation distance comparison.

It can be observed in Figure 10 that all of the routing schemes follow the same decreasing trend in end-to-end delay. This is because in a sparse network, there are less potential neighbors. On the other hand, in a dense region, a node finds more potential neighbors, which is beneficial for the reduction of propagation delay. This eventually reduces end-to-end delay. End-to-end delay primarily comprises propagation delay, holding time, transmission delay and calculation time. Accumulated propagation distance increases while considering two hops for routing decision in 2hop-AHH-VBF. That is why 2hop-AHH-VBF bears more end-to-end delay as compared with AHH-VBF and WDFAD-DBR. Up to node Number 350, 2hop-AHH-VBF follows the same trend as AHH-VBF and WDFAD-DBR. After that as the node number increases, the number of hops taken to reach the destination also increases. Holding time for packets is also introduced in both schemes. Successful transmissions towards the destination cause transmission delay, which is also added to overall end-to-end calculations along with holding time. In AHH-VBF, qualified node selection criteria are based on the distance from the virtual vector. Without considering residual energy and two-hop information, this causes packets to forward towards the void region. Transmission failure increases end-to-end delay due to retransmissions of the same packet. Therefore, in the QF-2hop-AHH-VBF scheme, selection of the qualified node and holding time calculation are based on the composite function as in Equation (10). This reduces the propagation delay due to avoidance of the overage hole and efficiently minimizes holding time. Hence, QF-2hop-AHH-VBF performs 30% better than AHH-VBF scheme. Similarly, utilizing the two-hop information along with the node position with respect to the virtual vector results in less hop traversing in QF-2hop-AHH-VBF. Hence, it performs 28% more efficient in reducing APD as compared to the AHH-VBF scheme as shown in the Figure 11.

## 6. Trade-Off Discussion

In this section, we discuss comparative analysis of the performance parameters of the proposed schemes with respect to the AHH-VBF scheme tabulated in Table 4. The proposed scheme 2hop-AHH-VBF performs better than the AHH-VBF scheme in PDR and energy tax, whereas end-to-end delay increases because of the increase in the propagation distance of the packet. 2hop-AHH-VBF has a void hole avoidance mechanism that secures high PDR compared to the AHH-VBF scheme. Energy tax reduces due to less packet drops. The packet forwarding decision based on two-hop information has reduced void hole probability, which increases the PDR and energy tax of the 2hop-AHH-VBF scheme. In the QF-2hop-AHH-VBF scheme, the forwarder is selected on the basis of the composite function including multiple parameters. Efficient forwarder selection improves PDR, which is 5.6% more than the AHH-VBF scheme. Holding time calculations in QF-2hop-AHH-VBF reduce end-to-end delay. Similarly, APD has also minimized end-to-end delay. Due to the aforementioned reasons, end-to-end delay and APD in QF-2hop-AHH-VBF are reduced by 28.5% and 28%, respectively. Energy tax increases due to a greater number of successful transmissions. Hence, end-to-end delay and PDR are improved at the cost of energy tax.

## 7. Conclusions

To improve the performance of sparse UWSNs, the 2hop-AHH-VBF protocol tackles the void hole problem. Routing decisions are made on the basis of neighbor node distribution in the ATD. Two-hop neighbor information before forwarding the data packet reduces the probability of void holes. The relative distance of the forwarding node and the neighbor information leads to optimal forwarder selection, which improves PDR. The simulation results validate that the performance of 2hop-AHH-VBF scheme beats the AHH-VBF routing scheme in terms of energy consumption and PDR. Energy per dropped packet is saved as the packet delivery is improved at the cost of end-to-end delay. While compared with the WDFAD-DBR and AHH-VBF counter routing techniques, 2hop-AHH-VBF performs efficiently, even in sparse UWSNs.

## Figures and Tables

**Table 1:** Summarized related works. H2-DAB, hop by hop dynamic addressing-based; EE, energy-efficient; WDFAD, weighting depth and forwarding area division; Re-Intar, reliable and interference-aware routing; VBF, vector-based forwarding; HH, hop by hop; AHH, adaptive hop by hop.

Techniques	Features	Parameters Achieved	Limitations
Proactive routing protocols (OLSR, DSDV, etc.) [5,6]	Routing tables are updated regularly to maintain routing information	Routes are always available	Large signaling overhead for establishing routing tables
Reactive routing protocols (AODV, DSR, etc.) [7,8]	Route discovery, Route maintenance	Bandwidth efficient, on demand approach	Higher latency, energy consumption
Beacon-based routing protocols such as H2-DAB [9]	Without location information, unique ID assignment to every node based on hop counts from sink	Improved reliability	Infeasible for low node mobility speed, high network overhead
Clustering routing [10,11] in UWSNs	Cluster head selection based on residual energy or location, nodes report data to cluster heads	Communication overhead minimization, efficient network management	Longer end-to-end delay, poor performance in time critical applications
DBR [12], depth-based routing protocol for UWSNs	Greedy forwarder selection, constant forwarding region all of the time	Energy conservation, network lifetime maximization	Inappropriate void hole avoidance mechanism, inefficient forwarder selection
EE-DBR [13], depth-based routing protocol for UWSNs	Energy conservation based routing protocol, depth-based routing protocol for UWSNs	Low end-to-end delay, energy tax	Inefficient void hole mechanism, large network overhead
WDFAD-DBR [14], depth-based routing protocol for UWSNs	Forwarder selection on the basis of two hop depth information, adaptive changes the forwarding area	Void hole avoidance, suppression of duplicate packets in local area network	Unnecessary energy consumption due to packet drop
Intar and Re-Intarrouting protocol [15], for UWSNs	One hop back transmission to avoid void hole, forwarder selection based on cost function	Void hole avoidance, high throughput	High accumulated propagation distance
VBF [16], location-based routing protocol for UWSNs	Confined forwarding area division to avoid packet duplication, directional forwarding along the virtual vector	High packet delivery ratio	Poor performance in sparse networks
HH-VBF [17], location-based routing protocol for UWSNs	Change in direction of pipeline hop-by-hop, directional forwarding	High network throughput	Inefficient void hole avoidance, poor performance in sparse networks
AHH-VBF [18], location-based routing protocol for UWSNs	Change in direction of pipeline, adaptive transmission range hop by hop	High network throughput, energy conservation	Inefficient forwarder selection

**Table 2:** Difference of terrestrial WSN (TWSN) and underwater wireless sensor network (UWSN).

TWSN	UWSN
Radio signal	Acoustic signal
High bandwidth	Low Bandwidth
Low propagation delay	High propagation delay
Low location error rate	High location error rate
High data rate	Low data rate

**Table 3:** Parameter setting.

Parameter	Value
Nodes	100–500 Random deployment
Sinks	1, on the surface
Network area	3D region of 10 km × 10 km × 10 km
Max power for transmission	90 dB re μ Pa
Max power for reception	10 dB re μ Pa
Max Transmission range	2000 m
Initial energy of each node	100 Joule
Node mobility	1–3 m/s
Data rate	16 kbps
Data packet size	111 bytes
Neighbor request packet size	66 bytes
Acknowledgment packet size	114 bytes
Center frequency	12 kHz
Bandwidth	4 kHz
Mobility model	Random walk mobility mode

**Table 4:** Comparative analysis of the performance parameters with respect to the AHH-VBF scheme.

Performance Parameters	2hop-AHH-VBF Scheme	QF-2hop-AHH-VBF Scheme	WDFAD-DBR Scheme
PDR (%)	2	5.6	2
Energy tax (%)	31.15	−45	−49.77
End-to-end delay (%)	−21	28.5	−56.13
APD (%)	−25	28	−53

## References

[1] Azam I., Javaid N., Ahmad A., Wadood A., Almogren A., Alamri A. (2017). Balanced Load Distribution with Energy Hole Avoidance in Underwater WSNs. IEEE Access.

[2] Javaid N., Shah M., Ahmad A., Imran M., Khan M.I., Vasilakos A.V. (2016). An Enhanced Energy Balanced Data Transmission Protocol for Underwater Acoustic Sensor Networks. Sensors.

[3] Akbar M., Javaid N., Khan A.H., Imran M., Shoaib M., Vasilakos A. (2016). Efficient data gathering in 3D linear underwater wireless sensor networks using sink mobility. Sensors.

[4] Larsson P. (2001). Selection diversity forwarding in a multihop packet radio network with fading channel and capture. ACM SIGMOBILE Mob. Comput. Commun. Rev..

[5] Clausen T., Jacquet P. (2003). Optimized Link State Routing Protocol (OLSR). http://www.rfc-editor.org/info/rfc3626.

[6] Perkins C.E., Bhagwat P. (1994). Highly dynamic destination-sequenced distance-vector routing (DSDV) for mobile computers. ACM SIGCOMM Computer Communication Review.

[7] Royer E.M., Perkins C.E. (1999). Multicast operation of the ad-hoc on-demand distance vector routing protocol. Proceedings of the 5th Annual ACM/IEEE International Conference on Mobile Computing and Networking.

[8] Johnson D.B., Maltz D.A. (1996). Dynamic source routing in ad hoc wireless networks. Mobile Computing.

[9] Ayaz M., Abdullah A. Hop-by-hop dynamic addressing based (H2-DAB) routing protocol for underwater wireless sensor networks. Proceedings of the International Conference on Information and Multimedia Technology.

[10] Domingo M.C. (2011). A distributed energy-aware routing protocol for underwater wireless sensor networks. Wirel. Pers. Commun..

[11] Liu G., Wei C. A new multi-path routing protocol based on cluster for underwater acoustic sensor networks. Proceedings of the 2011 International Conference on Multimedia Technology (ICMT).

[12] Yan H., Shi Z.J., Cui J.-H. (2008). DBR: Depth-based routing for underwater sensor networks. Proceedings of the International Conference on Research in Networking.

[13] Wahid A., Kim D. (2012). An energy efficient localization-free routing protocol for underwater wireless sensor networks. Int. J. Distrib. Sens. Netw..

[14] Yu H., Yao N., Wang T., Li G., Gao Z., Tan G. (2016). WDFAD-DBR: Weighting depth and forwarding area division DBR routing protocol for UASNs. Ad Hoc Netw..

[15] Majid A., Azam I., Khan T., Khan Z.A., Qasim U., Javaid N. A reliable and interference-aware routing protocol for underwater wireless sensor networks. Proceedings of the 2016 10th International Conference on Complex, Intelligent, and Software Intensive Systems (CISIS).

[16] Xie P., Cui J.-H., Lao L. (2006). VBF: Vector-based forwarding protocol for underwater sensor networks. Proceedings of the International Conference on Research in Networking.

[17] Xie P., Zhou Z., Nicolaou N., See A., Cui J.-H., Shi Z. (2010). Efficient vector-based forwarding for underwater sensor networks. EURASIP J. Wirel. Commun. Netw..

[18] Yu H., Yao N., Liu J. (2015). An adaptive routing protocol in underwater sparse acoustic sensor networks. Ad Hoc Netw..

[19] Sudevalayam S., Kulkarni P. (2011). Energy harvesting sensor nodes: Survey and implications. IEEE Commun. Surv. Tutor..

[20] Knight C., Davidson J., Behrens S. (2008). Energy options for wireless sensor nodes. Sensors.

[21] Syed A.A., Ye W., Heidemann J., Krishnamachari B. (2007). Understanding spatio-temporal uncertainty in medium access with ALOHA protocols. Proceedings of the Second Workshop on Underwater Networks.

[22] Peng Z., Zhu Y., Zhou Z., Guo Z., Cui J.H. COPE-MAC: A contention-based medium access control protocol with parallel reservation for underwater acoustic networks. Proceedings of the OCEANS 2010 IEEE Sydney.

